# An Ingested Foreign Body Performing a Vanishing Act

**DOI:** 10.14309/crj.0000000000000575

**Published:** 2021-05-11

**Authors:** Brian Park, Kiel Von Khan, Jaydeep Raval, Zachary Neubert, Ryan Fawley

**Affiliations:** 1 Department of Gastroenterology, Naval Medical Center San Diego, San Diego, CA; 2 Department of Internal Medicine, Naval Medical Center San Diego, San Diego, CA

## CASE REPORT

**Figure 1.**
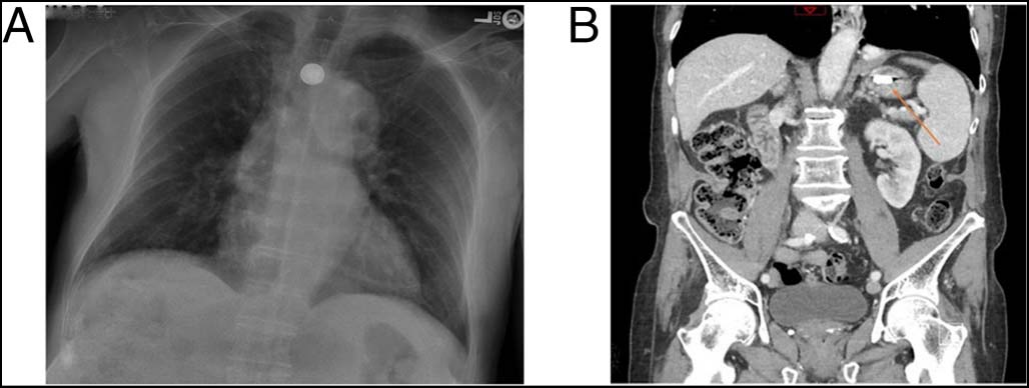
(A) Initial portable chest radiograph and (B) abdominal computed tomography revealed a 20-mm round, disc-like radiopaque foreign body within the middle 1/3 of the esophagus.

An 86-year-old man with chronic acid reflux, cryptogenic cirrhosis complicated by hepatic encephalopathy, ascites, and nonbleeding esophageal varices presented to the emergency department with progressive epigastric pain. A portable chest radiograph revealed a 20-mm, disc-like radiopaque foreign body within the middle one-third of the esophagus, which was seen migrating into the gastric fundus 1 hour later on sequential noncontrast abdominal computed tomography imaging (Figure 1). The patient denied foreign-body ingestion, including button battery or coin. Given the history of hepatic encephalopathy and the resemblance of a button battery on imaging, the patient underwent an emergent push upper endoscopy in adherence with guidelines from the American Society for Gastrointestinal Endoscopy on prompt removal of suspicious ingested foreign bodies.^1^ The foreign body was not found on endoscopy to the proximal jejunum. After procedure, follow-up radiographs no longer demonstrated the foreign body, and it seemed to have vanished.

**Figure 2.**
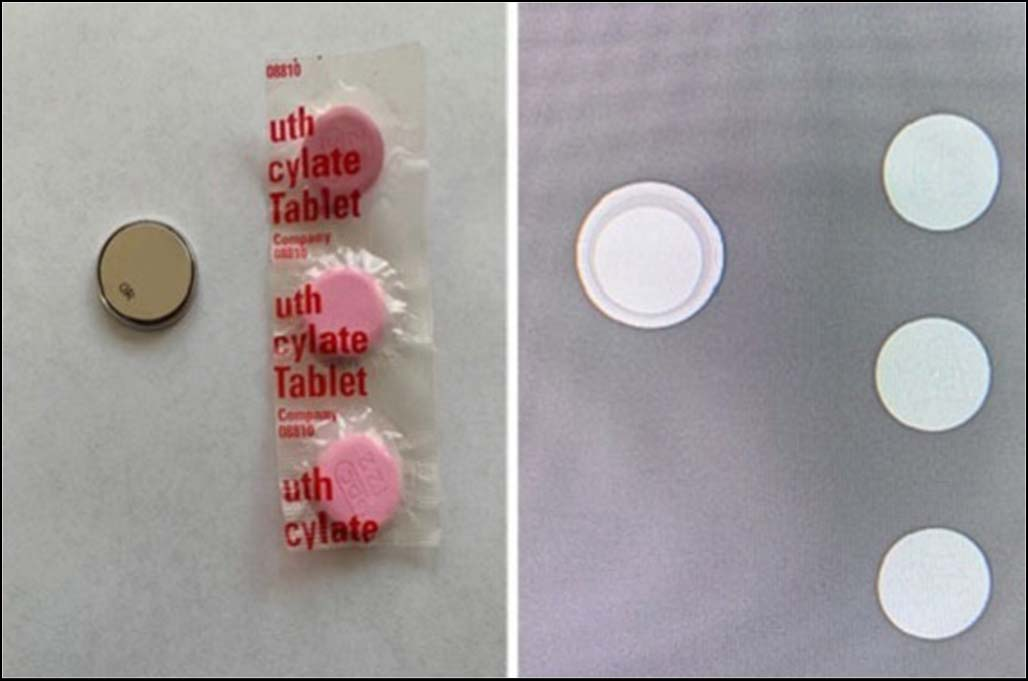
(A) Photograph of typical button battery vs bismuth subsalicylate tablet. (B) Fluoroscopic image of same button and whole bismuth subsalicylate tablet.

Further detailed review of the patient's medications identified tablets of bismuth salicylate with calcium carbonate, which the patient admitted to swallowing whole routinely, including just before presentation. Bismuth is notably radiopaque (Figure 2). We suspect that the tablet simply disintegrated in the stomach before the esophagogastroduodenoscopy based on the similar appearance of additional tablets he had on his person, detailed review of the computed tomography scan demonstrating subtle ill-defined edges of the ingested object not appreciated on initial chest radiographs, and absence of foreign body on the subsequent examination. The patient had an uneventful postendoscopic course and was safely discharged on a proton pump inhibitor for dyspepsia from the emergency department.

Some medications, including barium, lead, bismuth, iodine, and arsenic, are readily opaque on imaging and should be considered in evaluating vanishing radiopaque objects in the GI tract.^2–4^ This case highlights lessons learned of a rare situation in which ingesting whole tablets of bismuth subsalicylate can mimic a button battery, where a thorough history and knowledge of radiopaque medications could have mitigated the need for invasive procedures.

## DISCLOSURES

Author contributions: All authors contributed equally to this manuscript. B. Park is the article guarantor.

Financial disclosure: None to report.

Informed consent was obtained for this case report.
